# Pediatric Neck Swelling: A Case Report of Fourth Branchial Cleft Cyst

**DOI:** 10.7759/cureus.50149

**Published:** 2023-12-07

**Authors:** Noor I Al-Thawwab, Maryam J Alhashim, Ghaida S Alharbi, Kawkab M Alharbi, Ahmad A Abdultawab

**Affiliations:** 1 General Practice, Imam Abdulrahman Bin Faisal University, Dammam, SAU; 2 General Practice, Qassim University, Qassim, SAU; 3 General Practice, Princess Nourah Bint Abdul Rahman University, Riyadh, SAU; 4 Emergency Medicine, Dallah Hospital, Riyadh, SAU

**Keywords:** deep neck space infection, computed tomography, abscess, branchial cleft cyst, neck swelling

## Abstract

Pediatric neck masses present a diagnostic challenge, encompassing various etiologies, including rare entities like branchial cleft anomalies. Branchial cleft cysts, resulting from incomplete embryonic cleft obliteration, may become symptomatic. This case report describes a seven-year-old boy who presented with a week-long history of fever and progressively enlarging left anterior cervical swelling. Physical examination revealed a fluctuant, non-tender mass, prompting diagnostic investigations. Laboratory results indicated an elevated white blood cell count and inflammatory markers. Computed tomography identified a hypodense, rim-enhancing mass consistent with an abscess secondary to a fourth branchial cleft cyst. Ultrasound-guided aspiration yielded purulent material, confirming *Staphylococcus aureus* infection. This case highlights the clinical significance of fourth branchial cleft cysts as rare inflammatory neck masses in pediatric patients. The embryological context informs their diverse anatomical manifestations. Surgical excision remains the primary treatment, demanding consideration of anatomical complexities.

## Introduction

The occurrence of neck masses in pediatric patients poses a diagnostic challenge, necessitating a comprehensive understanding of diverse etiologies. Neck masses in children can arise from a myriad of causes, ranging from infectious to congenital anomalies. Among these, branchial cleft anomalies represent a rare but clinically significant subset. Branchial cleft cysts result from incomplete obliteration of embryonic branchial clefts during fetal development, leading to the formation of cystic structures that may later become symptomatic [1].

The diagnosis of branchial cleft cysts, in general, requires an understanding of the embryonic development of branchial clefts and pouches. These cysts develop from incomplete obliteration of these embryonic structures. This case describes a seven-year-old male who sought medical attention due to fever and a progressively enlarging neck swelling and was found to have a fourth branchial cleft cyst. While most branchial cleft cysts involve the second branchial arch, the fourth type stands out as an exceptional rarity [2]. The fourth branchial cleft cysts, following the course of the recurrent laryngeal nerve and predominantly located on the left side, present a unique diagnostic and therapeutic challenge due to their uncommon anatomical course and potential association with adjacent structures, such as the thyroid gland [1,2]. Careful consideration of such conditions is paramount for accurate diagnosis and successful management in pediatric cases [1].

## Case presentation

A seven-year-old male presented to the emergency department with a chief complaint of fever and neck swelling persisting for the past week. The parents reported a sudden onset of fever, reaching up to 101°F, associated with discomfort and pain in the neck region. The parents noticed a progressively enlarging swelling on the left side of the neck, prompting their visit to the hospital. The child had no significant past medical history, and there were no recent upper respiratory tract infections or trauma reported.

Upon physical examination, the patient appeared mildly distressed, with a temperature of 100.8°F, a heart rate of 110 beats per minute, and a respiratory rate of 20 breaths per minute. Inspection of the neck revealed a mass in the left anterior cervical region, measuring approximately 3 cm in diameter, with erythematous overlying skin. On palpation, the mass was tender and fluctuant.

Laboratory investigations were conducted, revealing an elevated white blood cell count of 15,000 cells/mm³, with a predominance of neutrophils. Inflammatory markers, including C-reactive protein, were significantly elevated. Blood cultures were obtained, and broad-spectrum intravenous antibiotics (cephalosporin and clindamycin) were initiated empirically.

In the context of the clinical findings, a broad differential diagnosis was considered, including infectious etiologies such as cervical lymphadenitis, deep neck space abscess, and branchial cleft anomalies. Non-infectious causes, such as neoplastic processes, were also considered, although less likely in the absence of constitutional symptoms and rapid onset.

**Figure 1:**
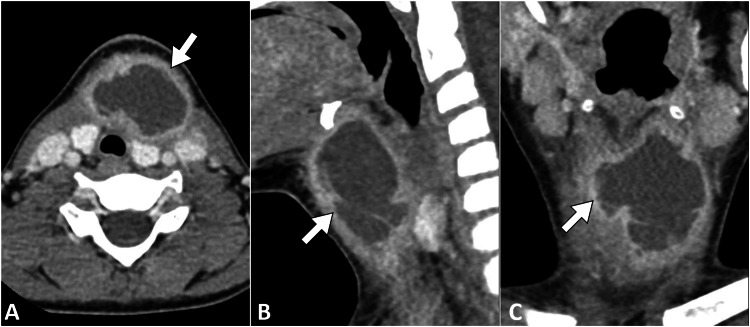
Enhanced CT images of the neck in axial (A), sagittal (B), and coronal (C) planes reveal a distinct collection (arrow) with an enhanced wall close to the thyroid gland. These findings are indicative of a fourth-type branchial cleft cyst. CT: computed tomography

A contrast-enhanced computed tomography scan of the neck was performed. The imaging demonstrated a hypodense mass lesion on the left side of the neck, measuring 2.5 cm in diameter. The lesion exhibited peripheral rim enhancement, consistent with an abscess formation. The adjacent structures, including the thyroid gland and major blood vessels, appeared unremarkable (Figure 1).

**Figure 2:**
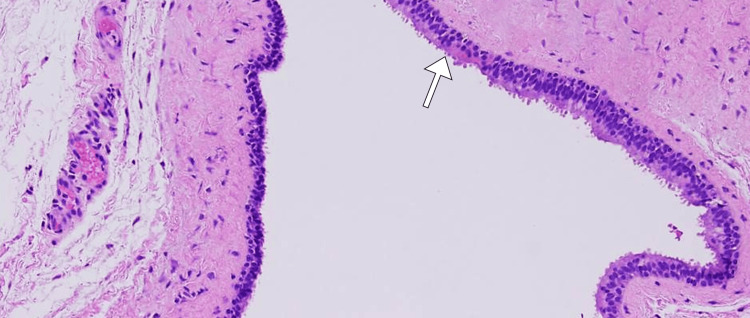
Histopathological examination showing cystic spaces lined by stratified squamous epithelium (arrow).

As part of the management strategy, the patient underwent ultrasound-guided aspiration of the neck mass for diagnostic purposes, and purulent material was successfully obtained. Microbiological cultures revealed the presence of *Staphylococcus aureus*, guiding targeted antibiotic therapy. Microscopic evaluation demonstrated a cystic structure lined by stratified squamous epithelium and the cystic wall exhibited fibrous tissue with occasional lymphoid aggregates (Figure 2). The final diagnosis, based on clinical, radiological, and microbiological findings, was determined to be an abscess secondary to a fourth branchial cleft cyst. Surgical intervention was subsequently planned for cyst excision and drainage to prevent recurrence.

The hospital course involved a multidisciplinary approach, including pediatric surgery, otolaryngology, and infectious disease specialists. The patient showed clinical improvement with appropriate antibiotic therapy and was afebrile within 48 hours. Surgical intervention was successfully carried out, with complete excision of the cystic structure.

Postoperatively, the patient demonstrated an uneventful recovery, and follow-up imaging confirmed the absence of residual or recurrent lesions. The patient was discharged with a course of oral antibiotics and scheduled for regular outpatient follow-up to monitor for any signs of recurrence or complications.

## Discussion

The differential diagnosis for pediatric neck masses is broad, encompassing infectious, congenital, and neoplastic etiologies. Branchial cleft anomalies represent a spectrum of developmental abnormalities, with second-branchial cleft lesions being the most common, while third- and fourth-branchial anomalies are notably rare, collectively accounting for approximately 20% of cervical masses in pediatric populations [2,3].

Clinical manifestations of fourth branchial cleft cysts often present as inflammatory neck masses, predisposing individuals to recurrent deep neck infections, such as acute suppurative thyroiditis [2]. The potential for recurrent infections and the development of fistula tracts pose challenges in management, with surgical intervention carrying the risk of injury to vital structures, particularly when the parotid is involved. The complexity of surgical considerations is underscored by the potential for facial nerve injury in cases involving the parotid region [3].

Embryologically, the branchial arches serve as precursors to a myriad of structures within the head, neck, and pharynx. The fourth branchial cleft, in particular, follows a unique course, originating from the lateral neck and looping across the aorta and subclavian artery before terminating near the cervical esophagus. This intricate embryological pathway explains the varied locations where fourth branchial cleft cysts may manifest, including the thyroid gland and mediastinum [4].

Management of branchial cleft anomalies involves making a precise diagnosis through careful history-taking, physical examination, and imaging studies. The surgical removal of the lesion is the primary modality of treatment, necessitating a tailored approach depending on the type and location of the cyst. Different surgical techniques, such as superficial parotidectomy and endoscopic cannulation, are employed to minimize the risk of complications, including facial nerve injury [2,5].

The inclusion of a less invasive option like sclerotherapy in the discussion adds depth to the management considerations, acknowledging that surgical intervention may not be feasible for all patients. The potential for infection further complicates the management algorithm, emphasizing the importance of timing in surgical intervention and the need for the resolution of acute infections before elective procedures [1,5].

## Conclusions

In conclusion, the presented case underscores the clinical significance of fourth branchial cleft cysts as rare types of inflammatory neck masses in pediatric patients. The embryological development of the branchial arches, particularly the fourth branchial cleft, illuminates the diverse anatomical structures associated with these anomalies. Surgical excision remains the mainstay of treatment, with careful consideration of anatomical complexities and potential complications. This case emphasizes the importance of a nuanced understanding of both embryological development and clinical aspects to navigate the diagnostic and management challenges posed by fourth branchial cleft cysts.
